# Substitution Effect of a Single Nitrogen Atom on π-Electronic Systems of Linear Polycyclic Aromatic Hydrocarbons (PAHs): Theoretically Visualized Coexistence of Mono- and Polycyclic π-Electron Delocalization

**DOI:** 10.3390/molecules29040784

**Published:** 2024-02-08

**Authors:** Jong Min Lim, Sangdeok Shim, Hoa Thi Bui, Jimin Kim, Ho-Joong Kim, Yoon Hwa, Sung Cho

**Affiliations:** 1 Department of Chemistry, Kyungpook National University, Daegu 41566, Republic of Korea; jongmin@knu.ac.kr; 2 Department of Chemistry, Sunchon National University, Suncheon 57922, Republic of Korea; san90@scnu.ac.kr; 3 Department of Chemistry, Chonnam National University, Gwangju 61186, Republic of Korea; hoak52ahoahoc@gmail.com (H.T.B.); jiminkim@chonnam.ac.kr (J.K.); 4 Department of Chemistry, Chosun University, Gwangju 61452, Republic of Korea; hjkim@chosun.ac.kr; 5 School of Electrical, Computer and Energy Engineering, Arizona State University, Tempe, AZ 85287, USA

**Keywords:** polycyclic aromatic hydrocarbons, heteroatom effect, π-electron delocalization, aromaticity, π-conjugation

## Abstract

We theoretically investigated the nitrogen substitution effect on the molecular structure and π-electron delocalization in linear nitrogen-substituted polycyclic aromatic hydrocarbons (N-PAHs). Based on the optimized molecular structures and magnetic field-induced parameters of fused bi- and tricyclic linear N-PAHs, we found that the local π-electron delocalization of subcycles (e.g., mono- and bicyclic constituent moieties) in linear N-PAHs is preserved, despite deviation from ideal structures of parent monocycles. The introduction of a fused five-membered ring with a pyrrolic N atom (N-5MR) in linear N-PAHs significantly perturbs the π-electronic condition of the neighboring fused six-membered ring (6MR). Monocyclic pyrrole exhibits substantial bond length alternations, strongly influencing the π-electronic systems of both the fused N-5MR and 6MR in linear N-PAHs, depending on the location of shared covalent bonds. A fused six-membered ring with a graphitic N atom in an indolizine moiety cannot generate monocyclic π-electron delocalization but instead contributes to the formation of polycyclic π-electron delocalization. This is evidenced by bifurcated diatropic ring currents induced by an external magnetic field. In conclusion, the satisfaction of Hückel’s 4n + 2 rule for both mono- and polycycles is crucial for understanding the overall π-electron delocalization. It is crucial to consider the unique characteristics of the three types of substituted N atoms and the spatial arrangement of 5MR and 6MR in N-PAHs.

## 1. Introduction

Polycyclic aromatic hydrocarbons (PAHs) have been of great interest in the field of organic electronics for their uses in emitting fluorophores, sensors, transistors, and light-harvesting apparatus [1,2,3,4]. This is due to their unique electronic and optoelectronic properties, which can be effectively tuned by manipulating the number of fused aromatic rings and their geometrical arrangement [5,6,7]. The addition of fused aromatic moieties increases the total number of coupled π-electrons and can modify the π-conjugation pathway and π-electron delocalization, facilitated by the structural homology of the constituent aromatic rings [8]. Generally, with an increasing number of fused aromatic rings, π-electrons become more widely delocalized, causing energetic destabilization and stabilization of the highest occupied molecular orbital (HOMO) and lowest unoccupied molecular orbital (LUMO), respectively. This leads to a decrease in transition energies of the lowest singlet (S_1_) and triplet (T_1_) states. However, it is known that increasing the number of fused aromatic rings has a limit in reducing the HOMO-LUMO gap [9]. In addition, two-dimensional PAHs have a limited number of geometrical configurations due to their inherent nature. Therefore, alternative techniques are needed to control the electronic characteristics of PAHs more effectively, instead of relying on conventional methods such as increasing fused rings or changing geometrical arrangements.

**Scheme 1:**
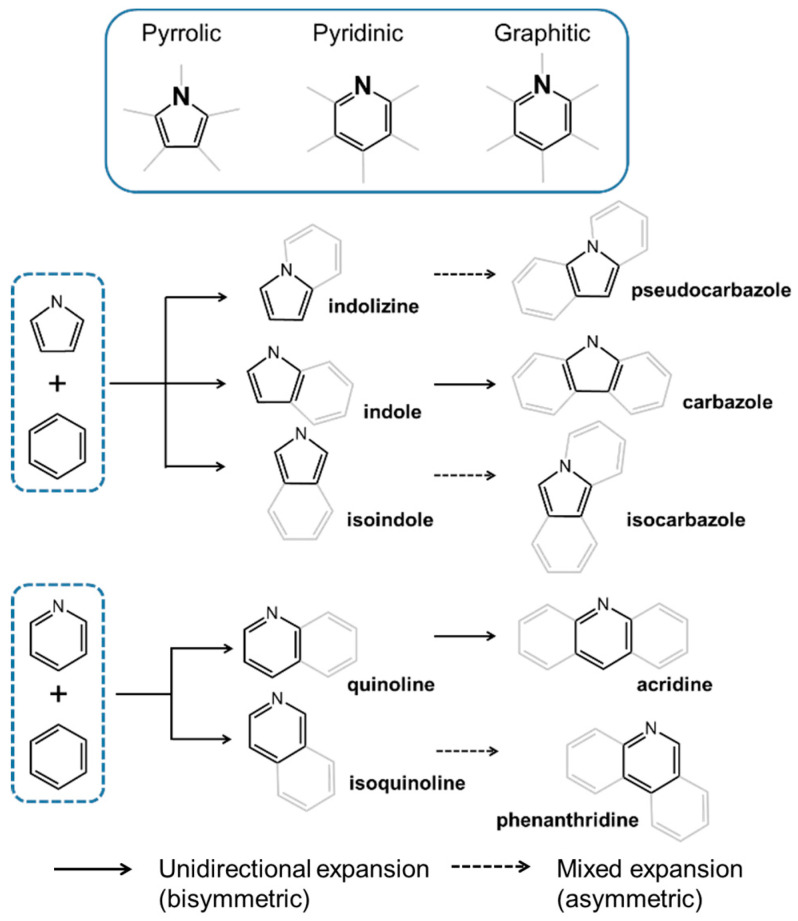
Expansion of N-substituted six- and five-membered aromatic rings with CH-hexagonal aromatic rings (the solid and dashed lines denote unidirectional and mixed expansion, respectively, depending on the location of shared bonds).

Nowadays, there have been various attempts to replace carbon atoms in PAHs with heteroatoms, such as nitrogen, sulfur, and oxygen atoms. This is performed to precisely adjust their structures and electronic properties similar to the use of dopants in silicon semiconductors [10,11,12,13,14,15]. Among these, nitrogen substitution has gained significant interest, as nitrogen-substituted PAHs (N-PAHs) are prevalent organic compounds, and several effective methods exist to dope nitrogen into graphene and PAHs, such as nitrogen plasma or chemical substitution [11,16,17,18]. There are at least three kinds of nitrogen-substituted six- and five-membered rings used as building blocks for N-PAHs with pyrrolic, pyridinic, and graphitic N atoms, which are widely found in popular organic compounds (Scheme 1) [19,20]. The five valence electrons of the nitrogen atom with the relatively high electron affinity can locally perturb π-electron distribution of the carbon-based PAHs. This raises an important issue of how the heteroatom effect of nitrogen atoms will impact the overall polycyclic (macroscopic) π-electron distribution and delocalization as it relates to the global electronic properties of N-PAHs.

We conducted a theoretical study on the linear N-PAHs that contain a mono-substituted nitrogen atom to investigate the effect of nitrogen atoms in PAHs. We utilized two types of N-substituted aromatic rings, pyridine and pyrrole, which were fused with six-membered aromatic hydrocarbon rings, as shown in Scheme 1. To avoid odd numbers of π-electrons and radicals in N-PAHs, we maintained a fixed number of covalent bonds at individual nitrogen atoms in five- and six-membered rings at three and two covalent bonds, respectively. Additionally, the linear N-PAHs are substituent-free because their π-electronic condition could be largely perturbed by peripheral substituents. As a result, there are three types of indole and carbazole isomers, as well as two types of quinoline and benzoquinoline isomers, respectively. The linear N-PAHs are employed to represent the three different types of local conditions of nitrogen atoms, namely pyridinic, pyrrolic, and graphitic nitrogen atoms. To investigate the π-electronic properties of the linear N-PAHs, we have employed various aromaticity indices based on optimized molecular structures. In indole and quinoline isomers, the π-electrons in the five- and six-membered rings with pyrrolic and pyridinic N atoms simultaneously contribute to both mono- and polycyclic π-electron conjugation, similar to six-membered aromatic hydrocarbons in naphthalene. On the other hand, the six-membered ring with a graphitic N atom in indolizine cannot cause monocyclic π-electron delocalization, and it seems to entirely contribute to the formation of polycyclic π-electron delocalization along the outward conjugation pathway that satisfies Hückel’s 4n + 2 rule. The six-membered rings with a graphitic N atom in indolizine and their expanded carbazole isomers exhibit low NICS(2) values, as well as bifurcated diatropic ring currents in the anisotropy of the induced current density (ACID) maps, indicating the absence of monocyclic π-electron delocalization and aromaticity. Conclusively, the number of π-electrons along the conjugation pathways and the satisfaction of Hückel’s 4n + 2 rule are critical factors in determining both mono- and polycyclic π-electron delocalization and the relative ratio of mono- and polycyclic π-electron distribution in the linear N-PAHs. The six-membered monocycle with a graphitic N atom cannot generate its own effective π-electron delocalization, which is obviously distinguished from the other aromatic polygons with pyrrolic and pyridinic N atoms.

## 2. Results and Discussion

### 2.1. Aromaticity Indices of Monocycles

In order to characterize the π-electronic systems of the linear N-PAHs, we have used various aromaticity indices to evaluate energetic, magnetic, and electronic properties, such as resonance energy and magnetic-induced ring current. Since linear N-PAHs consist of individual aromatic rings, we can examine the intricate changes in the π-electron environment by tracking alterations in the aromaticity indices of both individual constituent monocycles and extended polycycles. Comparing the relative aromaticity indices is useful for establishing the order of aromaticity through analog comparisons with similar molecular structures. However, it is worth noting that the aromaticity indices derived from optimized structures and those based on magnetic properties sometimes exhibit discrepancies. When comparing aromaticity indices of molecules with heteroatoms and/or different polygon structures, directly assigning the relative order of aromaticity and π-electron delocalization becomes challenging due to these inconsistencies in aromaticity indices. For instance, the structure-based HOMA and magnetic-based ACID critical isosurface values of monocycles exhibit a remarkably high correlation coefficient of 1.000 (Figure S2 in the Supplementary Materials). Moreover, the values for benzene (0.999, 0.082) and pyridine (1.000, 0.082) surpass those of pyrrole (0.872, 0.066), indicating that six-membered benzene and pyridine are more aromatic than five-membered pyrrole. However, the magnetic-based NICS(0) and NICS(1) values, which are widely employed to evaluate local aromaticity, do not align with the relative order observed in the HOMA and ACID values (Table 1). The highly negative NICS(0) and NICS(1) values are associated with a significant contribution from in-phase components [21], such as σ bonds and atoms around the NICS probe. Occasionally, we solely utilized the out-of-phase component of NICS to compare the strength of the induced diamagnetic ring current. Nevertheless, extracting the out-of-phase component necessitates additional computational effort and still does not align perfectly with the HOMA and ACID values. Despite both ACID and NICS calculations relying on the magnetic property of compounds, it remains challenging to establish the order of aromaticity.

**Figure 1:**
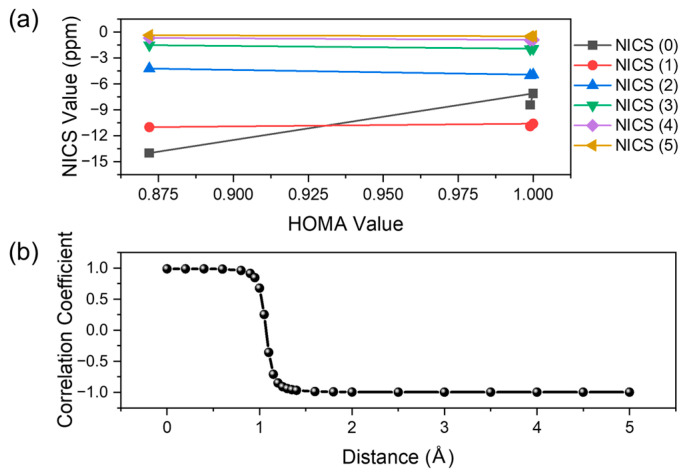
(**a**) Correlation between HOMA and NICS(x) values of parent monocycles and (**b**) correlation coefficients depending on the distance from the molecular mean plane.

We aimed to find a correlation between NICS and HOMA values based on the distance from the molecular mean plane of monocycles. We observed a significant negative correlation between NICS(2) and NICS(3) values with HOMA and ACID values (Figure 1). This negative correlation implies that the monocycles with higher HOMA values and more negative NICS values are more aromatic. Because the NICS(0) probe on the molecular mean plane in five-membered pyrrole is approximately 15.5% and 21.2% closer to the constituent atoms and covalent bonds, respectively, compared to six-membered benzene and pyridine (Figure S3 in the Supplementary Materials), the magnetic effects of the constituent atoms and covalent bonds in pyrrole are more significant. As Gershoni-Poranne et al. reported that NICS_πZZ_ plots at a height lower than 1.7 Å show σ-effect contaminations [22], the NICS probe in five-membered pyrrole should be positioned further away from the molecular mean plane to make a more accurate comparison of the strength of diatropic ring currents. We conclude that five-membered pyrrole is less aromatic and less effective for π-electron delocalization than six-membered benzene and pyridine. This is likely due to the smaller ring size and reduced symmetry of the five-membered polygon with a pyrrolic N atom, leading to highly concentrated π-electrons and substantially destabilized HOMO and HOMO-1 in comparison to benzene and pyridine. We utilized NICS(2) values to evaluate the strength of diamagnetic-induced ring currents in the linear N-PAHs as a polygon(shape)-independent index of local aromaticity and π-electron delocalization.

### 2.2. Visualized Diatropic Ring Currents of Bi- and Tricyclic N-PAHs

**Figure 2:**
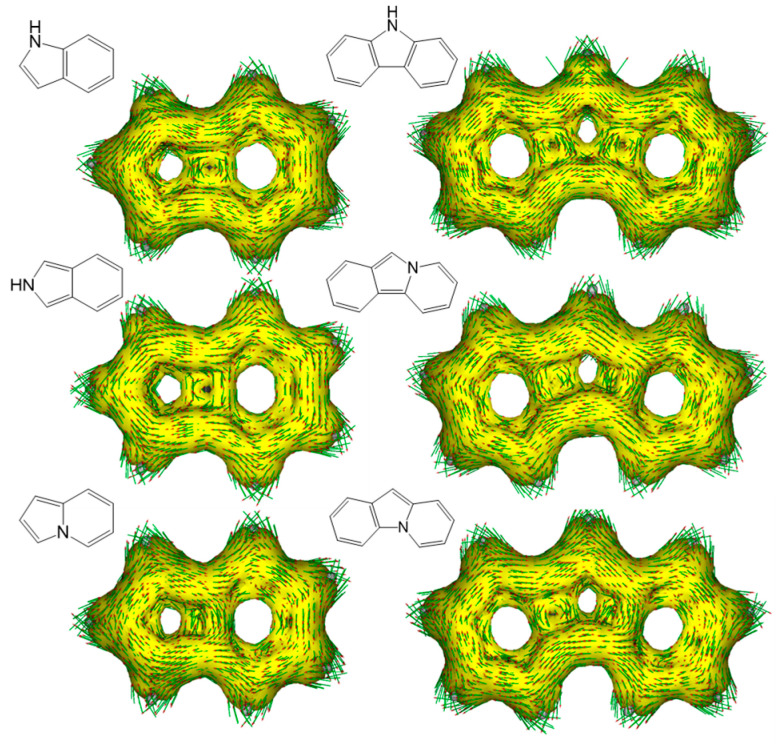
Magnetic-induced current maps of indole and carbazole isomers (clockwise vectors: diatropic ring current).

We investigated the effect of a substituted nitrogen atom in linear PAHs by comparing the aromaticity indices of bicyclic indole and quinoline isomers. To determine the extent of electron delocalization in fused bicyclic PAHs containing a single nitrogen atom, we theoretically visualized magnetic-induced current maps (Figure 2 and Figure S5 in the Supplementary Materials). With the exception of indolizine, the magnetic-induced ring currents of indole and quinoline isomers resembled that of naphthalene, exhibiting outward unidirectional diatropic ring currents (clockwise) characteristic of a conjugated 10-electron system. In contrast, indolizine displayed distinct bifurcated diatropic ring currents, with one flowing around the N-substituted five-membered ring and the other flowing outward (Figure 2). This bifurcated diatropic ring current observed in indolizine indicates the similar magnitudes of the two diatropic ring currents circulating the five-membered monocycle and the nine-membered polycycle, revealing the local- and poly/macrocyclic aromaticity, respectively. Notably, despite the fusion of rings, the N-substituted five-membered ring in indolizine maintained discernible pyrrolic characteristics.

To investigate whether the occurrence of bifurcated diatropic ring currents is common, we expanded the sample series to include linear tricyclic N-PAHs (Scheme 1). By visualizing induced ring current density maps, we confirmed that two carbazole isomers, isocarbazole and pseudocarbazole with an indolizine moiety, exhibit bifurcated diatropic ring currents (Figure 2 and Figure S6 in the Supplementary Materials). This observation suggests that bifurcated diatropic ring currents in linear N-PAHs with an indolizine moiety are not uncommon. We also noted that the presence of a constituent six-membered ring with a graphitic N atom in linear N-PAHs particularly promotes the formation of such bifurcated diatropic ring currents. The role of a graphitic N atom in the π-electron delocalization of PAHs will be discussed in the subsequent section.

### 2.3. How to Quantify the Bifurcated Diatropic Ring Currents

To evaluate the bifurcated diatropic ring currents, we compared the HOMA and NICS(2) values of indole and carbazole isomers. The HOMA values of the constituent five- and six-membered rings in indole and carbazole isomers were lower than those of their parent monocycles. However, their NICS(2) values generally increased, indicating increased diamagnetic ring currents due to extended π-conjugation and enriched π-electron delocalization caused by the fusion of rings (Table 2 and Table S1 in the Supplementary Materials). The structural deformation of the individual rings in indole and carbazole isomers is directly related to the formation of poly/macrocyclic π-electron conjugation and delocalization, which corresponds well with high HOMA values along outward polycyclic pathways, resembling 10- and 14π-electronic systems.

The π-conjugation pathways of individual constituent rings in the N-PAHs are spatially overlaid with the outward polycyclic conjugation pathways, making it challenging to accurately determine the monocyclic characteristics based on the structure-based HOMA values of the constituent rings alone, even with the selection of monocyclic covalent bonds. However, NICS(2) provides a means to assess the relative diatropic ring currents of the individual constituent rings, providing collective information about both mono- and polycyclic π-electronic systems at the assigned individual rings. To elucidate the distinct nature of the bifurcated diatropic ring currents in indolizine, we refocused on bicyclic indole and quinoline isomers. In bicyclic indole and quinoline isomers, there are only two possible π-conjugation pathways, monocyclic and outward bicyclic. Linear bicyclic N-PAHs form closed loops, resulting in equal inflow and outflow of polycyclic current density in neighboring constituent rings. If the NICS(2) values of individual constituent rings are polygon-independent, we can deduce that the differences of NICS(2) values between neighboring rings in indole isomers are attributable to varying strengths of monocyclic ring currents. This is because the contribution of the polycyclic ring current cancels out when calculating the difference of NICS(2) values. The nearly identical NICS(2) values of constituent rings in quinoline isomers, similar to those of naphthalene, indicate that substituting a carbon atom with a pyridinic nitrogen atom does not disrupt the mono- and polycyclic π-electronic systems. Conversely, the indole isomers exhibit larger differences in NICS(2) values by 6 to 12 times, suggesting heterogeneous π-electronic conditions for the individual constituent rings by the fusion between five- and six-membered rings (Table 2).

### 2.4. Structural Disparities between the N-5MR and C-6MR

The NICS(2) values of the C-6MR in indole and N-5MR in isoindole are higher than those of the neighboring polygonal N-5MR and C-6MR, respectively. Pino-Rios et al. proposed the Glidewell–Lloyd rule to explain the relatively larger aromaticity indices of 5MR, which prevent the formation of the smallest antiaromatic 4n group [23]. However, when using NICS(2) values to evaluate local aromaticity and π-electron delocalization, the higher NICS(2) value of the C-6MR in indole does not conform to the Glidewell–Lloyd rule. In this context, we need to consider the structural uniqueness of the parent N-5MR (pyrrole) and the location of the shared covalent bond between the N-5MR and 6MR. Although the fused bicyclic N-PAHs are distinct molecules compared to parent monocycles, the constituent 5MR and 6MR in N-PAHs likely retain their monocyclic characteristics, representing the concept of local aromaticity. If we assume that pyrrole is the ideal aromatic structure of N-5MR for the monocyclic π-electron delocalization, then we should compare the structural changes in the N-5MR in PAHs with the structure of pyrrole. Since pyrrole is not a regular pentagon, we can use this comparison to evaluate its monocyclic π-electron delocalization. The different locations of shared bonds in indole isomers result in heterogeneous effects on the combination of mono- and polycyclic π-electron delocalization. By examining the differing bond lengths of individual monocyclic rings in PAHs compared to parent monocycles, it is possible to evaluate the structural effect caused by the fusion of rings. For ease of use, we have chosen to use Pauling bond numbers derived from the lengths of C-C and C-N bonds to measure the structural deviation, as they allow for the comparison of π-electronic contribution for individual covalent bonds [24]. For instance, although the bond length of the shared covalent bond in naphthalene is 2.73% longer than that of benzene, its Pauling bond number is reduced by 15.1%, directly impacting the weakening of monocyclic (local) π-electronic characteristics and the introduction of additional polycyclic π-electron delocalization. Parent pyrrole has three types of covalent bonds with different Pauling bond numbers of 1.740, 1.314, and 1.398, which correspond to the shared covalent bond locations in indole, isoindole, and indolizine, respectively (Figure S7 in the Supplementary Materials). By comparing the Pauling bond numbers of shared covalent bonds in indole isomers with those of parent monocycles, we can evaluate the deviation of Pauling bond numbers individually for shared covalent bonds at the 6MR and 5MR, and the trend of reduced bond numbers aligns well with the trend of NICS(2) values. For instance, the Pauling bond numbers of shared covalent bonds in indole isomers are 1.330, 1.126, and 1.207 (Figure S8 in the Supplementary Materials). The Pauling bond numbers of shared covalent bonds are reduced by 23.6%, 14.3%, and 13.7% for N-5MR, and reduced by 15.2%, 28.2%, and 24.3% for 6MR in indole, isoindole, and indolizine, respectively. The 5MR in indole and the 6MRs in isoindole and indolizine are structurally more deviated from parent monocycles, and this correlates with the relative order of NICS(2) values for the individual constituent rings. The substantial decrease in Pauling bond numbers compared to parent monocycles indicates a discriminatively attenuated monocyclic 6π-electron delocalization and the emergence of newly formed polycyclic 10π-electron delocalization through the fusion of rings. Conclusively, the locations of shared covalent bonds in indole isomers are critical for the selection of relatively weak monocyclic π-electronic characteristics of the constituent N-5MR and 6MR.

### 2.5. Graphitic N Atom in Linear N-PAHs

The NICS(2) values of the constituent N-5MR and C-6MR in indole and isoindole differ by 0.88 and 0.78 ppm, respectively. In indolizine, the difference is approximately twice as large, 1.59 ppm. It is noteworthy that the NICS(2) value of the N-6MR in indolizine is significantly reduced compared to that in isoindole, despite having a larger HOMA value (Table 2). Interestingly, the N-6MR in indolizine with a graphitic N atom cannot satisfy Hückel’s 4n + 2 rule, which could be confirmed by localized π-electrons in a virtual six-membered monocycle with a graphitic N atom (Figure S7 in the Supplementary Materials). On the other hand, the outward polycyclic conjugation pathway in indolizine satisfies Hückel’s 4n + 2 rule, implying that the N-6MR in indolizine favors the formation of an outward polycyclic 10π-electronic system while minimally contributing to the monocyclic π-electronic system of the N-6MR. Conclusively, the N-5MR in indolizine contributes to both 6π monocyclic delocalization and 10π polycyclic delocalization with N-6MR, which manifests as bifurcated ring currents. The loss of monocyclic π-electronic characteristics of N-6MR in indolizine prevents collision between opposite monocyclic diatropic ring currents at the shared covalent bond. Similarly, the N-6MR with a graphitic N atom in isocarbazole and peudocarbazole cannot generate a monocyclic diatropic ring current, thereby leading to bifurcated diatropic ring currents of the 10- and 14π-electronic systems. The single N atom in an indolizine moiety simultaneously acts as a pyrrolic N atom in the N-5MR and a graphitic N atom in the N-6MR.

### 2.6. Coexistence of Mono- and Polycyclic π-Electronic Systems in Linear N-PAHs

**Scheme 2:**
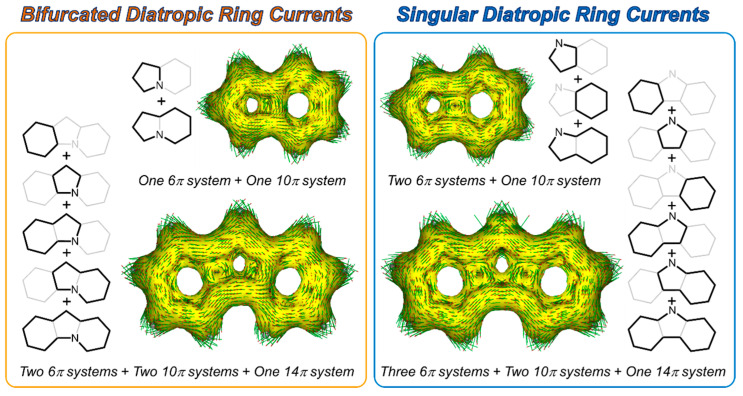
Coexistence of mono- and polycyclic π-electronic systems with bifurcated and singular diatropic ring currents.

When linear N-PAHs are expanded by fusing two or three constituent rings, a unique type of six-membered monocycle with a graphitic nitrogen atom is produced. This particular feature is present in isomers of indole and carbazole, which highlights local π-electronic characteristics of its neighboring mono- and (sub-)polycycles in linear N-PAHs. This demonstrates the coexistence of mono- and polycyclic electron delocalization in indolizine and the two carbazole isomers, shown as bifurcated diatropic ring currents (Scheme 2). It is important to note that fulfilling Hückel’s 4n + 2 rule is crucial for both constituent rings and fused polycycles with two or three rings to comprehend the overall electronic characteristics. The S_1_ and T_1_ states, which are the lowest excited states of linear N-PAHs, are primarily governed by the HOMO-LUMO transition. Therefore, the longest outward conjugation pathway that satisfies Hückel’s 4n + 2 rule is of paramount importance in explaining the representative electronic dynamics in these lowest excited states. Nevertheless, it is still fundamentally significant to understand the local characteristics exhibited by constituent monocycles and sub-polycycles with a smaller number of constituent rings, as overall thermodynamic stability and chemical reactivity are mainly determined by these local characteristics. Moreover, the relative contribution of π-electron delocalization along the longest conjugation path is also determined by these local characteristics. This is because the structural deformation for the longest conjugation path is constrained by the established local energetic benefits and delocalization by constituent mono- and (sub-)polycycles. Therefore, the polarizability and optical properties of the electrons should be considered based on the coexistence of mono- and polycyclic electronic systems in linear N-PAHs.

## 3. Materials and Methods

The molecular structures of linear PAHs and monocycles were optimized using the density functional theory (DFT) method with Becke’s three-parameter hybrid exchange functionals and the Lee–Yang–Parr correlation functional (B3LYP), employing the 6-311G(2df,p) basis set [25,26,27]. The calculations were performed on a supercomputer (KISTI, Nurion, Daejeon, Korea). Various aromaticity indices were utilized to characterize the π-electrons in linear PAHs. Nuclear-independent chemical shifts (NICS(x)) were calculated to check the isotropic shielding by constituent atoms on the monocyclic π-conjugation pathways at x Å above the molecular planes [21]. We confirmed that the experimental NMR results of indole and indolizine were well matched with the calculated NMR results based on their optimized molecular structures with GIAO-B3LYP/6-311G(2df,p) (Figure S1 in the Supplementary Materials). To plot the anisotropy of the induced current density (ACID) maps, the continuous set of gauge transformations (CSGT) method was employed to calculate the magnetically induced current densities [28,29]. The results were visualized using POVRAY 3.7 for Windows [30]. In order to compare the structure-based aromaticity of the mono- and polycyclic rings in linear PAHs, the harmonic oscillator model of aromaticity (HOMA) values were calculated [31]. To establish a practical nomenclature for monocycles, we employed concise symbols, namely a six-membered ring (6MR), a five-membered ring (5MR), a carbon atom-exclusive cycle (C-), and a mono-nitrogen-atom-substituted cycle (N-). Therefore, benzene, pyridine, and pyrrole are denoted as C-6MR, N-6MR, and N-5MR, respectively.

## 4. Conclusions

We conducted a theoretical study on the substitution effect of a single nitrogen atom in linear bi- and tricyclic N-PAHs to better understand electron delocalization and bifurcated diatropic ring currents. Using various aromaticity indices for linear N-PAHs, we evaluated the magnetic-induced ring currents in these molecules by using NICS(2) values for individual constituent monocycles. We looked at the substitution effect of three types of N atoms, pyrrolic, pyridinic, and graphitic, taking into consideration both the polygon shape and the number of covalent bonds at the substituted N atom. Despite deviations from the ideal structures of parent monocycles, our analysis showed that the local electron delocalization of subcycles (such as mono- and bicyclic constituent moieties) in linear N-PAHs is preserved, resulting in a less effective electron delocalization within the subcycles, which is related to the emergence of extended global polycyclic π-electron delocalization. In the case of N-6MR with a graphitic N atom, it cannot solely generate monocyclic π-electron delocalization, but contributes to the formation of polycyclic electron delocalization, shown by the bifurcated diatropic ring currents. This work highlights the significance of the satisfaction of Hückel’s 4n + 2 rule for both sub- and macrocycles in comprehending the overall π-electron delocalization, as well as the optimized molecular structures and spatial distribution of π-electrons in linear N-PAHs. To understand the mono- and polycyclic electronic properties, it is crucial to consider the unique characteristics of the three types of substituted N atoms and the spatial arrangement of 5MR and 6MR in N-PAHs. This study of linear bi- and tricyclic N-PAHs will be a milestone for understanding the relationship between the molecular structure of extended N-PAHs and their π-electronic properties.

## Figures and Tables

**Table 1:** Aromaticity indices of aromatic monocycles.

Compound	HOMA	ACID Critical Isosurface Value	NICS(0)	NICS(1)	NICS(2)	NICS(3)
Benzene (C-6MR)	0.999	0.082	−8.44	−10.9	−5.01	−1.96
Pyridine (N-6MR)	1.000	0.082	−7.11	−10.6	−4.93	−1.93
Pyrrole (N-5MR)	0.872	0.066	−14.0	−11.0	−4.22	−1.51

**Table 2:** Aromaticity indices of aromatic bicycles.

Compound	Location	HOMA	ACID Critical Isosurface Value	NICS(2)
Naphthalene	C-6MR	0.812	0.076	−5.50
	C-10MR	0.857	0.085	
Indole	N-5MR	0.747	0.056	−4.51
	C-6MR	0.937	0.065	−5.39
	N-9MR	0.871	0.069	
Isoindole	N-5MR	0.787	0.078	−5.35
	C-6MR	0.679	0.072	−4.57
	N-9MR	0.865	0.079	
Indolizine	N-5MR	0.840	0.081	−5.30
	N-6MR	0.737	0.072	−3.71
	N-9MR	0.863	0.072	
Quinoline	N-6MR	0.845	0.072	−5.43
	C-6MR	0.822	0.070	−5.50
	N-10MR	0.877	0.084	
Isoquinoline	N-6MR	0.850	0.078	−5.42
	C-6MR	0.837	0.078	−5.55
	N-10MR	0.873	0.082	

## Data Availability

Data are contained within the article and Supplementary Materials.

## Supplementary Materials

The following supporting information can be downloaded at: https://www.mdpi.com/article/10.3390/molecules29040784/s1, Table S1. Aromaticity indices of the linear tricyclic N-PAHs; Figure S1: H-NMR comparisons between experimental and theoretical (GIAO-B3LYP/6-311G(2df,p)) results of indole and indolizine; Figure S2: Correlation between HOMA and ACID values of monocycles; Figure S3: Averaged distance from atoms and covalent bonds to the center (NICS probe); Figure S4: Magnetic-induced current maps of six- and five-membered monocycles (benzene, pyridine, and pyrrole); Figure S5: Magnetic-induced current maps of fused bicycles with constituent six- and five-membered rings; Figure S6: Magnetic-induced current maps of fused tricycles with constituent six- and five-membered rings; Figure S7: Bond lengths (black), Pauling bond numbers (red), and HOMA parameters of monocycles and N-6MRs with different charge states; Figure S8: Bond lengths (black), Pauling bond numbers (red), and HOMA parameters of bicycles; Figure S9: Bond lengths (black), Pauling bond numbers (red), HOMA parameters of tricycles, and optimized geometry of molecular structures.
