# Supplementary material for: Substitution Effect of a Single Nitrogen Atom on π-Electronic Systems of Linear Polycyclic Aromatic Hydrocarbons (PAHs): Theoretically Visualized Coexistence of Mono- and Polycyclic π-Electron Delocalization
Source: Molecules. 2024 Feb 8;29(4):784. doi: 10.3390/molecules29040784 (PMC10892997; doi:10.3390/molecules29040784)
Supplement: Supplementary file 1 [file molecules-29-00784-s001.zip › molecules-2845501-supplementary.pdf]

Supplementary Materials

Substitution Effect of a Single Nitrogen Atom on π-Electronic Systems of Linear Polycyclic Aromatic Hydrocarbons (PAHs); Theoretically Visualized Coexistence of Mono- and Poly-cyclic π-Electron Delocalization

Jong Min Lim [1,†], Sangdeok Shim [2,†], Hoa Thi Bui [3], Jimin Kim [3], Ho-Joong Kim [4], Yoon Hwa [5,*] and Sung Cho [3,*]

[1] Department of Chemistry, Kyungpook National University, Daegu 41566, Republic of Korea
[2] Department of Chemistry, Sunchon National University, Suncheon 57922, Republic of Korea
[3] Department of Chemistry, Chonnam National University, Gwangju 61186, Republic of Korea
[4] Department of Chemistry, Chosun University, Gwangju 61452, Republic of Korea
[5] School of Electrical, Computer and Energy Engineering, Arizona State University, Tempe, AZ 85287, USA

Table S1. Aromaticity indices of the linear tricyclic N-PAHs.

Compound	Location	HOMA	AICD critical isosurface value	NICS(2)
Anthracene	Center C-6MR	0.760	0.081	- 6.69
	Side C-6MR	0.662	0.072	- 5.21
	C-10MR	0.792	0.081	
	C-14MR	0.806	0.082	
Phenanthrene	Center C-6MR	0.511	0.076	- 4.94
	Side C-6MR	0.891	0.077	- 5.44
	C-10MR	0.698	0.076	
	C-14MR	0.779	0.081	
Acridine	Center N-6MR	0.765	0.074	- 6.57
	Side C-6MR	0.664	0.069	- 5.19
	N-10MR (quinoline)	0.797	0.074	
	N-14MR	0.811	0.084	
Phenanthridine	Center N-6MR	0.629	0.075	- 4.89
	C-6MR (C-6MR')	0.902 (0.912)	0.072 (0.079)	- 5.38 (- 5.48)
	N-10MR (N-10MR)'	0.769 (0.762)	0.075 (0.075)	
	N-14MR	0.824	0.075	
Carbazole	Center N-5MR	0.657	0.061	- 4.31
	Side C-6MR	0.958	0.069	- 5.26
	N-9MR (indole)	0.826	0.061	
	N-13MR	0.891	0.078	
Isocarbazole	Center N-5MR	0.725	0.071	- 5.95
	Side C-6MR	0.801	0.072	- 5.25
	Side N-6MR	0.803	0.074	- 4.21
	N-9MR (isoindole)	0.842	0.076	
	N-9MR' (indolizine)	0.839	0.071	
	N-13MR	0.885	0.074	
Pseudocarbazole	Center N-5MR	0.714	0.061	- 5.37
	Side C-6MR	0.917	0.071	- 5.62
	Side N-6MR	0.686	0.073	- 3.31
	N-9MR (indole)	0.845	0.078	
	N-9MR (indolizine)	0.772	0.061	
	N-13MR	0.845	0.073	

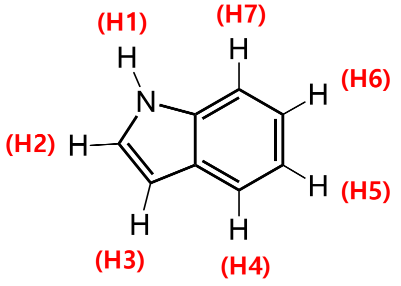

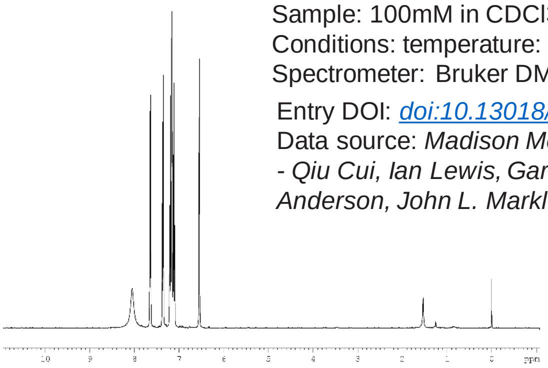

Sample: 100mM in CDCl3, ref: TMS
Conditions: temperature: 298K, pH: n/a
Spectrometer: Bruker DMX - 400MHz

Entry DOI: *doi:10.13018/BMSE000097*
Data source: *Madison Metabolomics Consortium - Qiu Cui, Ian Lewis, Gareth Westler, Mark E. Anderson, John L. Markley*

	Experiment (NMR)		B3LYP/6-311G(2df, p)	
	Indolizine in $CDCl_3$		Indolizine w/ scrf = CCl_4	
H position	Chemical Shift (ppm)	relative	Chemical Shift (ppm)	relative
**H3**	6.48	0.00	7.26	0.00
**H5**	7.05	0.57	7.88	0.62
**H2**	7.10	0.62	7.90	0.64
**H6**	7.13	0.64	7.93	0.68
**H7**	7.30	0.82	8.06	0.80
**H4**	7.58	1.10	8.35	1.10
**H1**			8.51	1.25

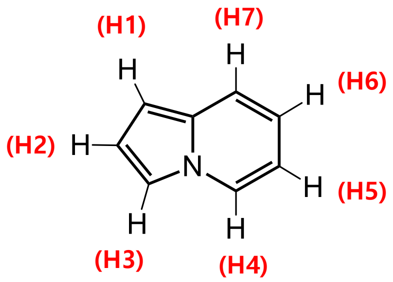

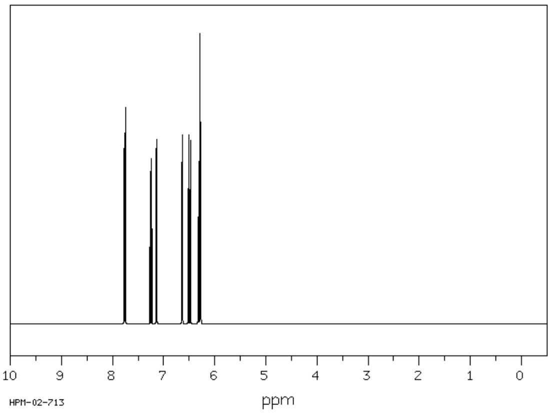

	Experiment (NMR)		B3LYP/6-311G(2df, p)	
	Indolizine in CCl4		Indolizine w/ scrf = CCl4	
H position	Chemical Shift (ppm)	relative	Chemical Shift (ppm)	relative
**H1**	6.28	0.00	7.14	0.00
**H5**	6.31	0.03	7.20	0.05
**H6**	6.50	0.22	7.34	0.20
**H2**	6.64	0.36	7.54	0.40
**H3**	7.14	0.86	7.98	0.83
**H7**	7.25	0.97	8.03	0.89
**H4**	7.76	1.48	8.60	1.45

Entry DOI: doi.org/10.1071/CH9641128
Black, P.; Heffernan, M.; Jackman, L.; Porter, Q.; Underwood, G., Proton Magnetic Resonance Spectra of Indolizine and Its Methyl Derivatives and Aza Analogues. *Australian Journal of Chemistry* **1964**, *17*, 1128-1137.

Figure S1. H-NMR comparisons between experimental and theoretical (GIAO-B3LYP/6-311G(2df,p)) results of indole and indolizine.

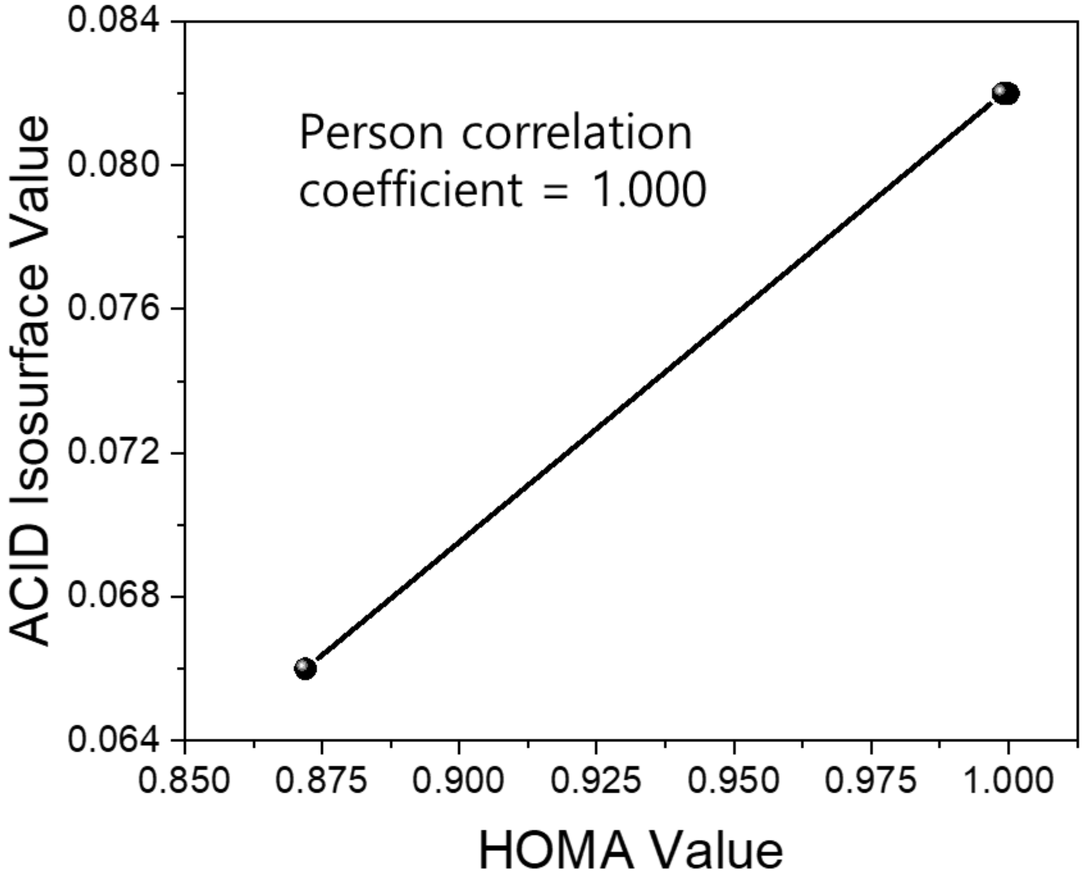

Figure S2. Correlation between HOMA and ACID values of monocycles.

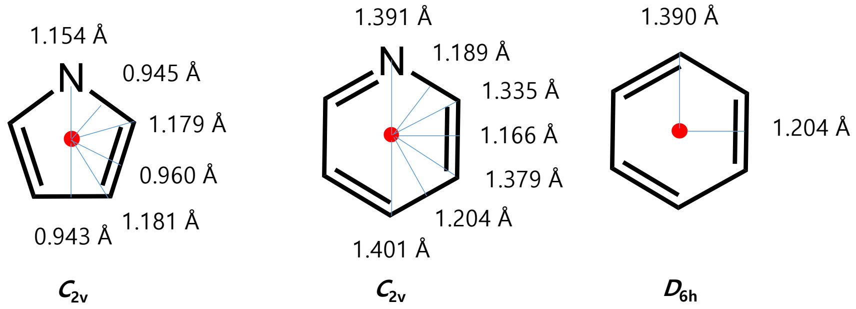

	Averaged Distance (reduced distance compared to those of benzene)		
**center – atom**	1.175 Å (- 15.5 %)	1.370 Å (- 1.44 %)	1.390 Å
**center – bond**	0.949 Å (- 21.2 %)	1.186 Å (- 1.47 %)	1.204 Å

Figure S3. Averaged distance from atoms and covalent bonds to the center (NICS probe).

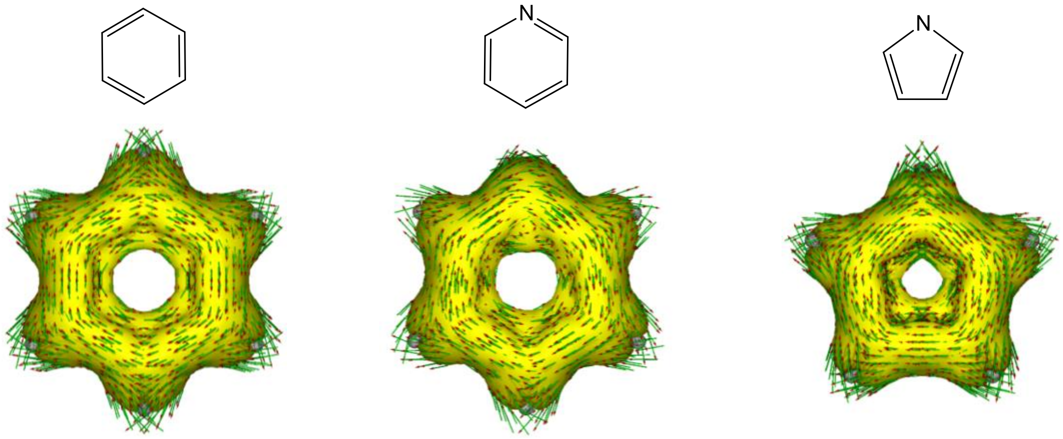

Figure S4. Magnetic induced current maps of six- and five-membered monocycles; benzene, pyridine, and pyrrole.

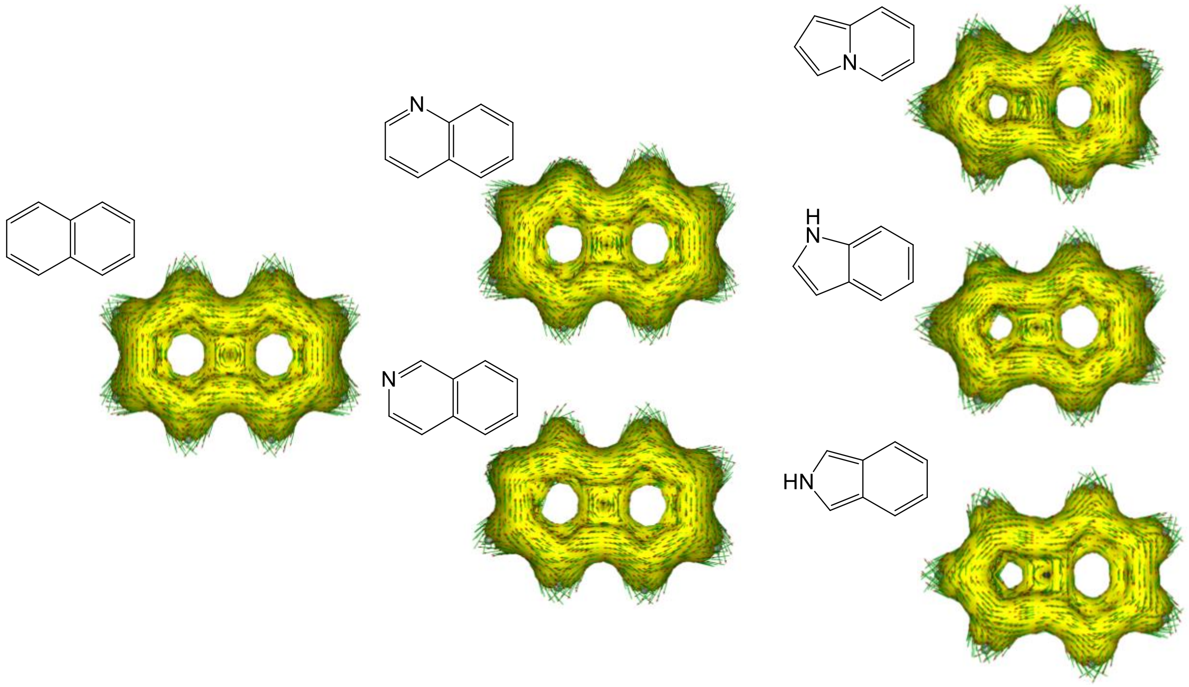

Figure S5. Magnetic induced current maps of fused bicycles with constituent six- and five-membered rings**.**

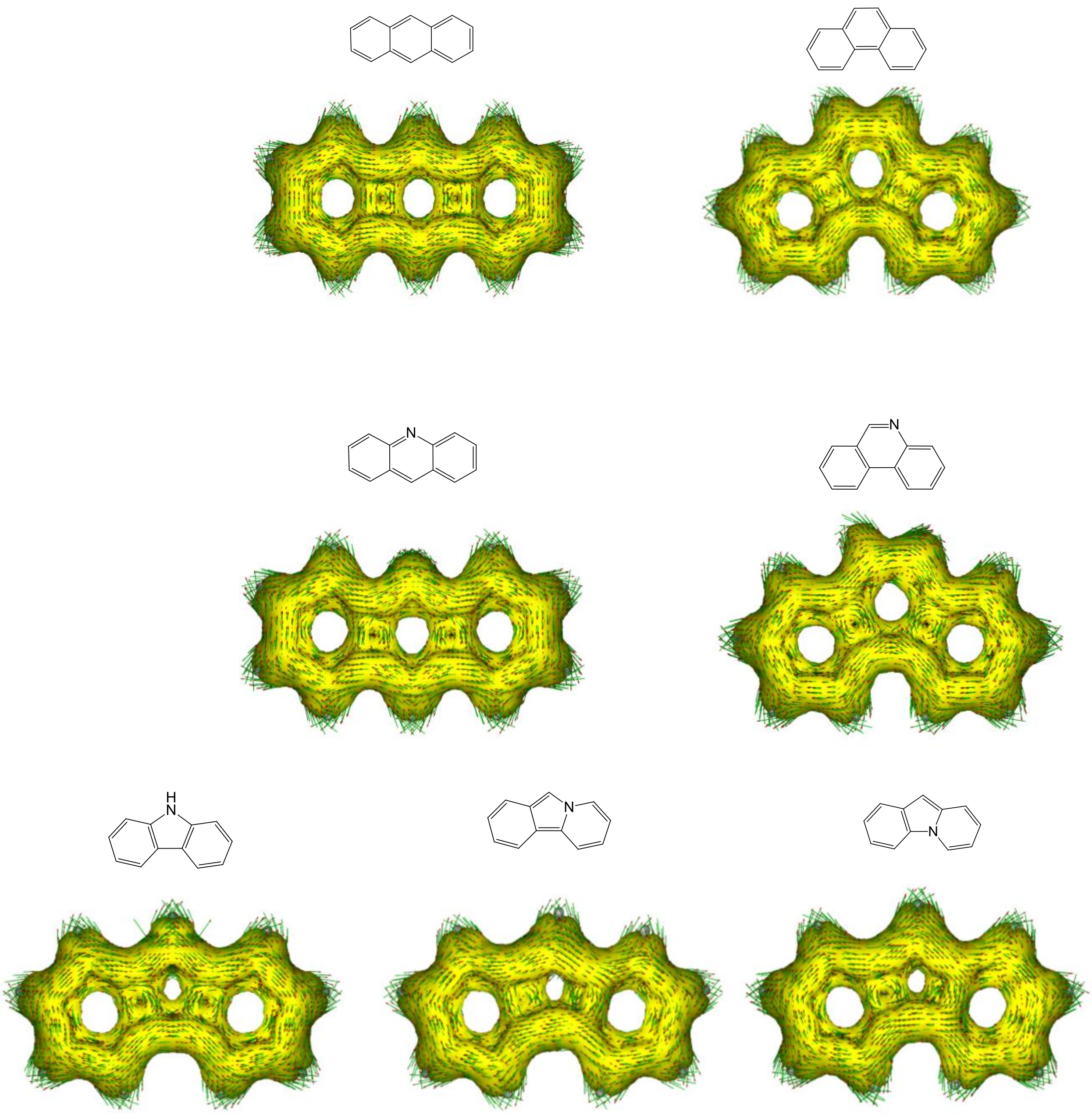

Figure S6. Magnetic induced current maps of fused tricycles with constituent six- and five-membered rings.

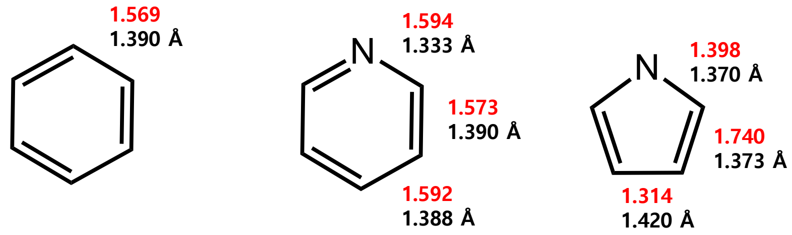

E	0.001
G	0.000
H	0.999

E	0.000
G	0.000
H	1.000

E	0.022
G	0.107
H	0.872

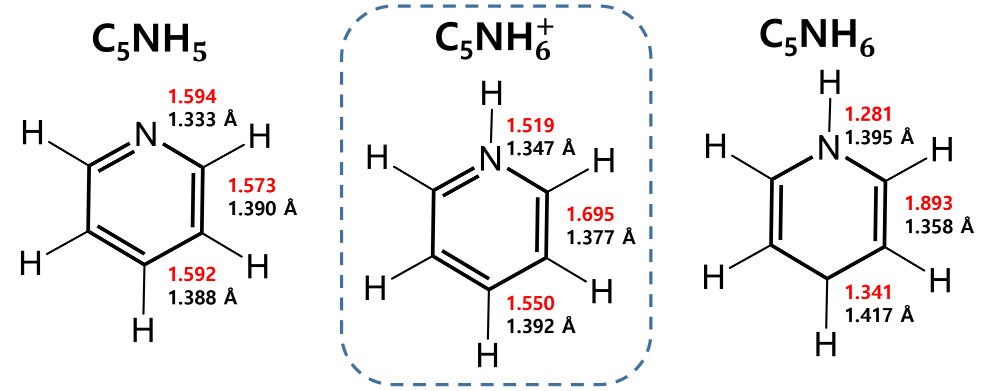

E	0.000
G	0.000
H	1.000

E	0.000
G	0.017
H	0.983

E	0.038
G	0.227
H	0.735

Figure S7. Bond lengths (black), Pauling bond numbers (red), and HOMA parameters of monocycles and N-6MRs with different charge states.

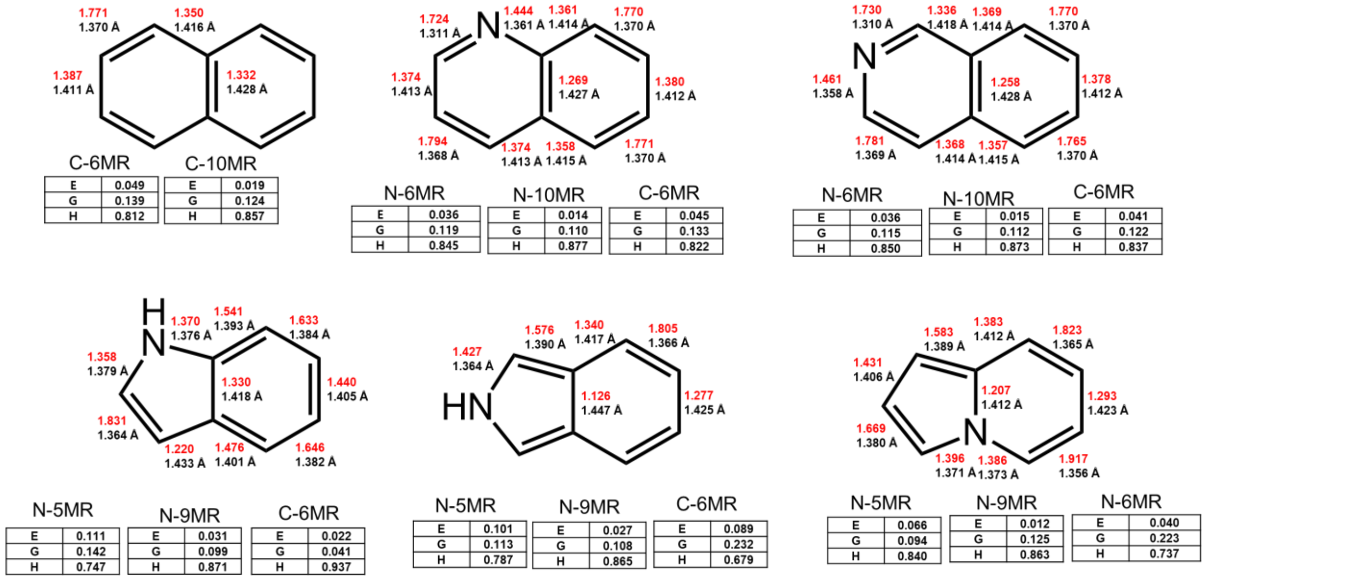

Figure S8. Bond lengths (black), Pauling bond numbers (red), and HOMA parameters of bicycles.

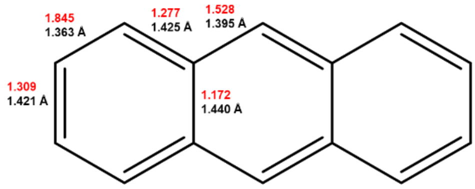

Side C-6MR

E	0.086
G	0.252
H	0.662

Center C-6MR

E	0.123
G	0.117
H	0.760

C-10MR

E	0.048
G	0.159
H	0.792

C-14MR

E	0.027
G	0.167
H	0.806

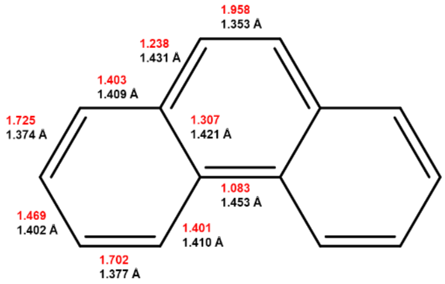

Side C-6MR

E	0.030
G	0.079
H	0.891

Center C-6MR

E	0.237
G	0.252
H	0.511

C-10MR

E	0.083
G	0.218
H	0.698

C-14MR

E	0.041
G	0.180
H	0.779

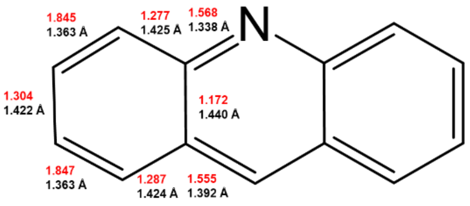

Side C-6MR

E	0.085
G	0.252
H	0.664

Center N-6MR

E	0.098
G	0.137
H	0.765

N-10MR

E	0.038
G	0.165
H	0.797

N-14MR

E	0.021
G	0.168
H	0.811

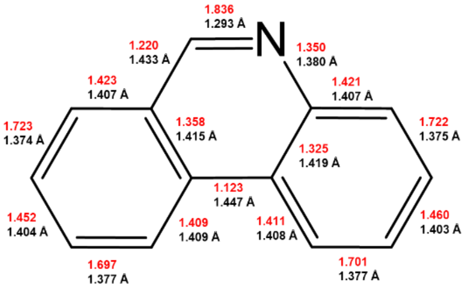

Side C-6MR′ (isoquinoline)

E	0.024
G	0.065
H	0.912

Center N-6MR

E	0.197
G	0.173
H	0.629

Side C-6MR (quinoline)

E	0.026
G	0.072
H	0.902

N-10MR′

E	0.074
G	0.164
H	0.762

N-14MR

E	0.037
G	0.139
H	0.824

N-10MR

E	0.070
G	0.162
H	0.769

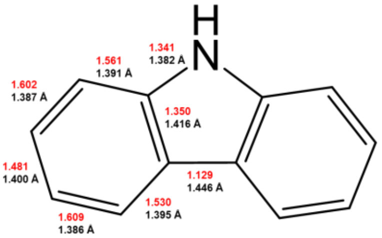

Side C-6MR

E	0.016
G	0.027
H	0.958

Center N-5MR

E	0.306
G	0.037
H	0.657

N-9MR

E	0.085
G	0.090
H	0.826

N-13MR

E	0.036
G	0.073
H	0.891

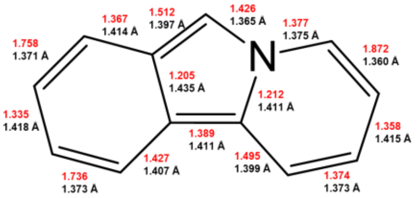

Side C-6MR

E	0.057
G	0.142
H	0.801

Center N-5MR

E	0.213
G	0.061
H	0.725

Side N-6MR′

E	0.031
G	0.166
H	0.803

N-9MR (isoindole)

E	0.061
G	0.096
H	0.842

N-13MR

E	0.019
G	0.096
H	0.885

N-9MR′ (indolizine)

E	0.043
G	0.118
H	0.839

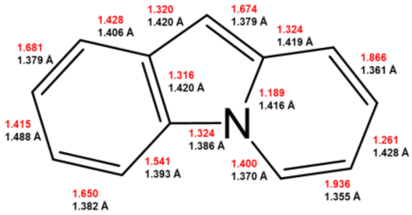

Side C-6MR

E	0.026
G	0.057
H	0.917

Center N-5MR

E	0.191
G	0.095
H	0.714

Side N-6MR′

E	0.047
G	0.267
H	0.686

N-9MR (indole)

E	0.055
G	0.099
H	0.845

N-13MR

E	0.020
G	0.135
H	0.845

N-9MR′ (indolizine)

E	0.045
G	0.183
H	0.772

Figure S9. Bond lengths (black), Pauling bond numbers (red), and HOMA parameters of tricycles.

Optimized molecular structures (B3LYP/6-311G(2df,p))

1. Benzene

C	0.00000000	1.39029300	0.00000000
C	1.20402900	0.69514700	0.00000000
C	1.20402900	-0.69514700	0.00000000
C	0.00000000	-1.39029300	0.00000000
C	-1.20402900	-0.69514700	0.00000000
C	-1.20402900	0.69514700	0.00000000
H	0.00000000	2.47396700	0.00000000
H	2.14251800	1.23698300	0.00000000
H	2.14251800	-1.23698300	0.00000000
H	0.00000000	-2.47396700	0.00000000
H	-2.14251800	-1.23698300	0.00000000
H	-2.14251800	1.23698300	0.00000000

2. Pyridine

C	0.00000000	1.34663400	0.00000000
C	1.20338100	0.65109200	0.00000000
C	1.16549400	-0.73623900	0.00000000
C	-0.07105600	-1.36633200	0.00000000
C	-1.21608600	-0.57845000	0.00000000
N	-1.19443900	0.75449800	0.00000000
H	2.08157400	-1.31498200	0.00000000
H	-0.00486700	2.43267300	0.00000000
H	2.14424000	1.18748900	0.00000000
H	-0.15134500	-2.44637300	0.00000000
H	-2.19893400	-1.04053100	0.00000000

3. Pyrrole

C	0.00000000	1.12107100	0.33031700
C	0.00000000	0.71022800	-0.97944800
C	0.00000000	-0.71022800	-0.97944800
C	0.00000000	-1.12107100	0.33031700
N	0.00000000	0.00000000	1.11812300
H	0.00000000	0.00000000	2.12293700
H	0.00000000	2.10771300	0.76213100
H	0.00000000	1.35651300	-1.84224400
H	0.00000000	-1.35651300	-1.84224400
H	0.00000000	-2.10771300	0.76213100

4. Naphthalene

C	0.00000000	2.42309100	0.70569000
C	0.00000000	1.24048000	1.39682000
C	0.00000000	0.00000000	0.71401900
C	0.00000000	0.00000000	-0.71401900
C	0.00000000	1.24048000	-1.39682000
C	0.00000000	2.42309100	-0.70569000
H	0.00000000	-1.23905900	2.48130900
H	0.00000000	3.36521800	1.24076600
H	0.00000000	1.23905900	2.48130900
C	0.00000000	-1.24048000	1.39682000
C	0.00000000	-1.24048000	-1.39682000
H	0.00000000	1.23905900	-2.48130900
H	0.00000000	3.36521800	-1.24076600
C	0.00000000	-2.42309100	-0.70569000
C	0.00000000	-2.42309100	0.70569000
H	0.00000000	-1.23905900	-2.48130900
H	0.00000000	-3.36521800	-1.24076600
H	0.00000000	-3.36521800	1.24076600

5. Quinoline

C	2.38831100	-0.71426200	0.00000200
C	1.20213300	-1.39944600	0.00000100
C	-0.02855900	-0.70218100	0.00000000
C	-0.01403400	0.72426400	0.00000000
C	1.22800700	1.40189400	0.00000100
C	2.40290600	0.69781100	0.00000200
H	3.32658300	-1.25610300	0.00000300
H	1.16626700	-2.48144800	0.00000200
C	-1.26135500	1.38791800	-0.00000100
H	1.23447600	2.48648100	0.00000100
H	3.35067400	1.22252800	0.00000200
C	-2.41317500	0.65072500	-0.00000100
C	-2.31748300	-0.75897500	-0.00000300
H	-1.28927100	2.47223900	-0.00000100
H	-3.38696100	1.12432400	0.00000000
H	-3.22570400	-1.35656500	0.00000000
N	-1.18522400	-1.41970600	-0.00000100

6. Isoquinoline

C	-2.41813700	-0.58713700	0.00000000
C	-1.28463600	-1.35443100	0.00000000
C	-0.01877300	-0.72519700	0.00000000
C	0.00000000	0.69706000	0.00000000

C	-1.24760100	1.37033300	0.00000000
H	1.20252500	-2.50818700	0.00000000
H	-3.39912600	-1.05012800	0.00000000
H	-1.35328900	-2.43622600	0.00000000
C	1.21157000	-1.42415800	0.00000000
C	1.23833200	1.37864800	0.00000000
H	-1.25571600	2.45874700	0.00000000
C	2.41379700	0.67523400	0.00000000
C	2.39721900	-0.73710900	0.00000000
H	1.24241500	2.46300200	0.00000000
H	3.36255400	1.19787600	0.00000000
H	3.33556100	-1.27894100	0.00000000
N	-2.41222200	0.77062700	0.00000000

7. Indole

C	-0.24816200	0.74847600	0.00000800
C	-0.24691500	-0.67015000	-0.00004600
C	0.93216100	-1.41277000	0.00004800
C	2.12725900	-0.71564300	0.00019400
C	2.15048800	0.68906800	0.00024800
C	0.97942000	1.42327600	0.00015400
C	-1.62011900	1.16257700	-0.00004900
C	-2.38131900	0.03077600	-0.00032300
H	0.91688000	-2.49668100	0.00000200
H	3.06240200	-1.26255300	0.00027200
H	3.10522000	1.20111100	0.00037600
H	1.00910900	2.50684600	0.00022000
H	-1.99288800	2.17403100	0.00001500
H	-3.45326000	-0.08301600	-0.00049600
H	-1.87593800	-2.03152900	-0.00029500
N	-1.56120000	-1.07740900	-0.00021500

8. Isoindole

C	0.24975800	-0.72342300	0.00002000
C	0.24975800	0.72342300	-0.00005100
C	-0.98140900	1.42544500	0.00003300
C	-2.14718100	0.71267800	0.00018100
C	-2.14718100	-0.71267800	0.00024800
C	-0.98140900	-1.42544500	0.00016800
C	1.57818400	-1.13075800	-0.00004900
C	1.57818400	1.13075800	-0.00018600
H	-0.99433900	2.50950100	-0.00001000
H	-3.09699400	1.23441000	0.00025800

H	-3.09699400	-1.23440900	0.00037800
H	-0.99433800	-2.50950100	0.00023700
H	2.02421400	-2.11046300	-0.00001400
H	3.34843800	0.00000000	-0.00050300
H	2.02421500	2.11046300	-0.00026800
N	2.34193800	0.00000000	-0.00032400

9. Indolizine

C	0.27902100	0.74972500	-0.00005600
C	-0.96914100	1.40972900	0.00001700
C	-2.13062000	0.69311000	0.00015300
C	-2.08629900	-0.72937300	0.00022700
C	-0.89469100	-1.37699000	0.00016500
C	1.57390700	-1.10785100	-0.00002300
C	2.40225100	-0.00433300	-0.00039200
C	1.60723700	1.15523800	-0.00013000
H	-0.97692100	2.49239600	-0.00001600
H	-3.08736900	1.19875200	0.00022700
H	-2.99856600	-1.30988600	0.00035600
H	-0.79472000	-2.45345200	0.00023800
H	1.79676200	-2.16135400	0.00005800
H	3.48023600	-0.04208800	-0.00062300
H	1.94707100	2.17788900	-0.00015900
N	0.27764600	-0.66254200	0.00002200

10. Anthracene

C	0.00000000	3.64559900	0.71056700
C	0.00000000	2.47083400	1.40133700
C	0.00000000	1.21884400	0.71996700
C	0.00000000	1.21884400	-0.71996700
C	0.00000000	2.47083400	-1.40133700
C	0.00000000	3.64559900	-0.71056700
C	0.00000000	0.00000000	1.39819100
C	0.00000000	0.00000000	-1.39819100
C	0.00000000	-1.21884400	-0.71996700
C	0.00000000	-1.21884400	0.71996700
C	0.00000000	-2.47083400	1.40133700
H	0.00000000	-2.47008600	2.48570900
C	0.00000000	-3.64559900	0.71056700
C	0.00000000	-3.64559900	-0.71056700
C	0.00000000	-2.47083400	-1.40133700
H	0.00000000	0.00000000	2.48346900
H	0.00000000	4.58968600	1.24211600

H	0.00000000	2.47008600	2.48570900
H	0.00000000	2.47008600	-2.48570900
H	0.00000000	4.58968600	-1.24211600
H	0.00000000	0.00000000	-2.48346900
H	0.00000000	-4.58968600	1.24211600
H	0.00000000	-4.58968600	-1.24211600
H	0.00000000	-2.47008600	-2.48570900

11. Phenanthrene

C	0.00000000	3.54759100	-0.29495400
C	0.00000000	2.82699400	0.87505700
C	0.00000000	1.41772600	0.86247000
C	0.00000000	0.72671700	-0.37958200
C	0.00000000	1.49587600	-1.56077800
C	0.00000000	2.87194100	-1.52291900
C	0.00000000	0.67631100	2.08597900
C	0.00000000	-0.72671700	-0.37958200
C	0.00000000	-1.41772600	0.86247000
C	0.00000000	-0.67631100	2.08597900
C	0.00000000	-2.82699400	0.87505700
H	0.00000000	-3.33721400	1.83195700
C	0.00000000	-3.54759100	-0.29495400
C	0.00000000	-2.87194100	-1.52291900
C	0.00000000	-1.49587600	-1.56077800
H	0.00000000	1.22683700	3.02014900
H	0.00000000	4.63062600	-0.27086200
H	0.00000000	3.33721400	1.83195700
H	0.00000000	1.00466900	-2.52422700
H	0.00000000	3.43485200	-2.44866000
H	0.00000000	-1.22683700	3.02014900
H	0.00000000	-4.63062600	-0.27086200
H	0.00000000	-3.43485200	-2.44866000
H	0.00000000	-1.00466900	-2.52422700

12. Acridine

C	0.00000000	3.62639200	-0.65450300
C	0.00000000	2.47796300	-1.38792000
C	0.00000000	1.20718500	-0.74528100
C	0.00000000	1.15124600	0.69367800
C	0.00000000	2.37763900	1.42021500
C	0.00000000	3.57325800	0.76626000
C	0.00000000	0.00000000	-1.43818200
C	0.00000000	-1.15124600	0.69367800

C	0.00000000	-1.20718500	-0.74528100
C	0.00000000	-2.47796300	-1.38792000
H	0.00000000	-2.51318100	-2.47183900
C	0.00000000	-3.62639200	-0.65450300
C	0.00000000	-3.57325800	0.76626000
C	0.00000000	-2.37763900	1.42021500
H	0.00000000	0.00000000	-2.52390500
H	0.00000000	4.58957100	-1.15020000
H	0.00000000	2.51318100	-2.47183900
H	0.00000000	2.31415400	2.50081700
H	0.00000000	4.49903500	1.32932800
H	0.00000000	-4.58957100	-1.15020000
H	0.00000000	-4.49903500	1.32932800
H	0.00000000	-2.31415400	2.50081700
N	0.00000000	0.00000000	1.37534300

13. Phenanthridine

C	3.53032600	-0.24372300	-0.00000100
C	2.78556200	0.91164900	0.00012400
C	1.37931200	0.86058200	0.00000700
C	0.72062700	-0.39640000	-0.00010000
C	1.51083100	-1.56215400	-0.00026200
C	2.88547700	-1.48932600	-0.00022700
C	-0.72670400	-0.40373400	-0.00003700
C	-1.38575900	0.84825300	-0.00001100
C	-0.58824300	2.03908300	0.00002200
C	-2.79128200	0.91004900	0.00004800
H	-3.27310000	1.88151500	0.00005200
C	-3.53935700	-0.24295700	0.00009900
C	-2.89227700	-1.48839800	0.00012600
C	-1.51774600	-1.56917100	0.00006100
H	4.61247100	-0.19514200	0.00006800
H	3.24825900	1.89027800	0.00024800
H	1.03644300	-2.53489200	-0.00047400
H	3.47205700	-2.40013700	-0.00037400
H	-1.10261000	2.99972700	0.00011600
H	-4.62130500	-0.19574800	0.00014300
H	-3.48187300	-2.39746300	0.00021900
H	-1.04776400	-2.54346000	0.00013500
N	0.70468900	2.06468600	0.00011000

14. Carbazole

C	0.00000000	3.03344600	-1.14825600

C	0.00000000	3.41552500	0.19877300
C	0.00000000	2.47262600	1.21577400
C	0.00000000	1.12973500	0.85248400
C	0.00000000	0.72320300	-0.50378400
C	0.00000000	1.69408000	-1.50499200
H	0.00000000	3.79568300	-1.91755500
H	0.00000000	4.46868300	0.45302300
H	0.00000000	2.77500400	2.25648700
H	0.00000000	1.40608100	-2.54981600
C	0.00000000	-1.12973500	0.85248400
C	0.00000000	-2.47262600	1.21577400
C	0.00000000	-3.41552500	0.19877300
C	0.00000000	-3.03344600	-1.14825600
C	0.00000000	-1.69408000	-1.50499200
C	0.00000000	-0.72320300	-0.50378400
H	0.00000000	-2.77500400	2.25648700
H	0.00000000	-4.46868300	0.45302300
H	0.00000000	-3.79568300	-1.91755500
H	0.00000000	-1.40608100	-2.54981600
N	0.00000000	0.00000000	1.64884800
H	0.00000000	0.00000000	2.65379000

15. Isocarbazole

C	2.68601100	-1.79225500	0.00000000
C	1.81059100	-2.90758700	0.00000000
C	0.44848700	-2.75161500	0.00000000
C	-0.08752700	-1.44331800	0.00000000
C	0.80132700	-0.31631700	0.00000000
C	2.19442600	-0.51024000	0.00000000
H	3.75591000	-1.96086500	0.00000000
H	2.23443600	-3.90511400	0.00000000
H	-0.20751600	-3.61429300	0.00000000
H	2.87009200	0.33753300	0.00000000
C	-2.37286400	1.31198500	0.00000000
C	-2.11142600	2.64690600	0.00000000
C	-0.77915300	3.12346300	0.00000000
C	0.25461000	2.22044700	0.00000000
C	0.00000000	0.84518800	0.00000000
H	-3.36962100	0.89478200	0.00000000
H	-2.94415800	3.33734200	0.00000000
H	-0.58348700	4.18704700	0.00000000
H	1.28517500	2.55226800	0.00000000
H	-2.34305000	-1.47271700	0.00000000

C	-1.39725900	-0.95846900	0.00000000
N	-1.34015900	0.40498300	0.00000000

16. Pseudocarbazole

C	2.90068300	-1.20991700	0.00009400
C	3.39016400	0.13114000	0.00002500
C	2.52330900	1.18003200	-0.00003800
C	1.12205000	0.95468400	-0.00004400
C	1.56578200	-1.44023100	0.00007600
H	1.13253100	-2.42988500	0.00012100
C	-1.13604900	0.95025200	0.00000100
C	-2.51898000	1.20563000	0.00001100
C	-3.39928100	0.14463300	-0.00000100
C	-2.94254000	-1.18707200	-0.00002800
C	-1.59089500	-1.47452000	-0.00004500
C	-0.70155500	-0.40183500	-0.00001800
H	-2.88497500	2.22561800	0.00002100
H	-4.46613300	0.33421100	0.00000800
H	-3.66103100	-1.99725200	-0.00003800
H	-1.24540400	-2.50109000	-0.00006500
C	0.01647400	1.77943200	-0.00002200
N	0.68405600	-0.39191900	-0.00002600
H	0.04315200	2.85692200	-0.00005400
H	3.58279100	-2.04818200	0.00016700
H	2.87735100	2.20284700	-0.00007400
H	4.45835300	0.30687800	0.00003000
